# Protective Effects of Vitamin K Compounds on the Proteomic Profile of Osteoblasts under Oxidative Stress Conditions

**DOI:** 10.3390/molecules25081990

**Published:** 2020-04-23

**Authors:** Marta Muszyńska, Ewa Ambrożewicz, Agnieszka Gęgotek, Grzegorz Grynkiewicz, Elżbieta Skrzydlewska

**Affiliations:** 1 Department of Analytical Chemistry, Medical University of Bialystok, 15-222 Bialystok, Poland; marta.muszynska@umb.edu.pl (M.M.); ewaa.ambrozewicz@gmail.com (E.A.); agnieszka.gegotek@umb.edu.pl (A.G.); 2 Łukasiewicz Research Network, Pharmaceutical Research Institute, 01-793 Warsaw, Poland; g.grynkiewicz@ifarm.eu

**Keywords:** osteoblasts, proteomic analysis, vitamins K

## Abstract

Oxidative stress, which accompanies the pathogenesis of many bone diseases, contributes to the reduction of osteoblast activity, resulting in the inhibition of differentiation. This study aimed to assess the effect of vitamins K1 and K2 (MK4 and MK7) on the proteomic profile of human osteoblasts cell line under oxidative conditions induced by hydrogen peroxide (H_2_O_2_). The analysis was performed using QExactiveHF mass spectrometer with a nanoelectrospray ionization source. The osteoblast protein exposed to oxidative stress and vitamin K was compared with the proteome of cells exposed only to oxidative stress. Our proteomic analysis identified 1234 proteins changed after 5 days, 967 after 15 days, and 1214 after 20 days of culture. We observed the most frequent changes in the expression of proteins with catalytic activity or protein/DNA binding properties (45% and 40%, respectively). Significant changes were also observed in proteins with transcription/translation regulator activity (2–6%), regulators of molecular functions (5–6%), signal transducers (1–4%), transporters (4–6%), and structural molecules (3–5%). Our results clearly show that vitamins K protect cells from H_2_O_2_-induced changes in protein expression, primarily through their effects on transcriptional regulators and transporter proteins. As a result, vitamins K can support the formation, remodeling, and mineralization of bone tissue.

## 1. Introduction

Osteoblasts are osteogenic cells responsible for the production of components of organic bone tissue and its mineralization. The physiological and pathophysiological metabolism of osteoblasts is usually accompanied by the production of reactive oxygen species (ROS), the activity of which is balanced by the action of the antioxidant system [1]. However, the pathogenesis of many bone diseases associated with age, mechanical stress, and local inflammatory reactions may lead to increased production of ROS, such as peroxide anion, hydrogen peroxide (H_2_O_2_), nitric oxide, and peroxynitrite. Such disorders are also often associated with simultaneous disruption of the antioxidant system, including ROS removal mechanisms, leading to oxidative stress [2,3]. Oxidative conditions can cause the modification of macromolecules such as lipids, proteins, and DNA, altering transcriptional processes, gene expression, and cell signaling [4]. In osteogenic cells, oxidative stress causes metabolic changes, increasing the rate of degradation while reducing the potential for bone formation from osteoblasts and their precursors [5]. Numerous reports demonstrate a relationship between redox imbalance and increased bone resorption in the pathogenesis of metabolic bone diseases [6,7]. These changes are caused by a decrease in osteoblast activity accompanied by an increase in osteoclast activity, leading to a change in bone mineral density, which may cause weakening and greater susceptibility to fracture [8,9]. The decrease in osteoblast activity also inhibits the differentiation of these cells by reducing the synthesis of type I collagen, osteoprotegerin, sialoprotein, and osteocalcin, as well as alkaline phosphatase (ALP) activity [10,11,12].

Vitamin K compounds—specifically phylloquinone (K1) and menaquinones (K2: MK4, MK7)—have been identified as playing an important role in bone metabolism. Vitamin K (especially K2) has been shown to improve the metabolic functions of osteoblasts by inducing their proliferation, inhibiting apoptosis, and increasing the expression of osteogenic genes [13]. In agreement with their importance, vitamin K deficiencies are classified as a risk factor for bone disease, especially in the elderly [14].

The metabolic function of vitamin K is associated with post-translational γ-carboxylation of glutamate residues in various proteins. However under physiological conditions, vitamin K exists in the oxidized form (quinone), but γ-carboxylation of glutamate residues requires its reduced form (hydroquinone). In vivo, quinone is reduced by NAD(P)H and/or glutathione to hydroquinone, which next is converted to epoxide in the vitamin K redox cycle [15]. Thiol groups of proteins participate in epoxide reduction, under the influence of vitamin K epoxy reductase. Therefore, vitamin K is considered to be a factor regulating redox signaling in cells, as well as the activation of proteins involved in bone metabolism [16,17]. Moreover, data showing that ROS by increasing phospholipase A2 (PLA2) activity can induce arachidonic acid (AA) release [18,19], suggests that vitamin K by directly reducing the level of ROS [20] has also a modulatory effect on arachidonic acid cascade. Additionally, vitamin K can inhibit the activity of lipoxygenases (LOXs) [21]. LOX as an enzyme that metabolize arachidonic and linoleic acid is responsible for generation of signaling molecules, including prostaglandin E2 (PGE2), leukotriene B4 (LTB4), and hydroxyeicosatetraenoic acid (HETE) that are involved in the cell growth stimulation [22] or hydroxyoctadecadienoic acids (HODEs) found as cell growth and DNA synthesis inhibitor [23]. Moreover, PGE2 can additionally stimulate several cell signaling pathways such as extracellular signal-regulated kinase (ERK), P38α, and cAMP response element-binding protein (CREB) resulting in epithelial cancer cells growth acceleration [22]. Therefore, vitamin K by LOX inactivation can regulate cells growth under various conditions. In support of vitamin K’s protective effects is the observation that high levels of vitamin K correlate with a decrease in pro-inflammatory factors. This finding highlights the role of vitamin K in regulating the interaction of oxidative stress and inflammatory processes [24].

The regulatory role of vitamins K in osteoblast redox homeostasis means they can protect the structure and function of osteoblast cell proteins under conditions of oxidative stress. This protective effect may limit the development of bone diseases. Therefore this study aimed to uncover the effects of vitamins K1 and K2 (MK4 and MK7) on the osteoblast proteome during proliferation and differentiation under oxidative stress conditions induced by hydrogen peroxide (H_2_O_2_).

## 2. Results

In this study, we reported the effects of vitamin K1 and K2 (MK4 and MK7) treatment on protein expression in human fetal osteoblast cell line (hFOB 1.19) after exposure to hydrogen peroxide in vitro. As a result of the proteomic analysis, we identified, quantified, and assigned 1234 proteins after 5 days, 967 after 15 days, and 1214 after 20 days of culture The IDs of these proteins, as well as their levels in each group, are presented in Table 1.

**Figure 1:**
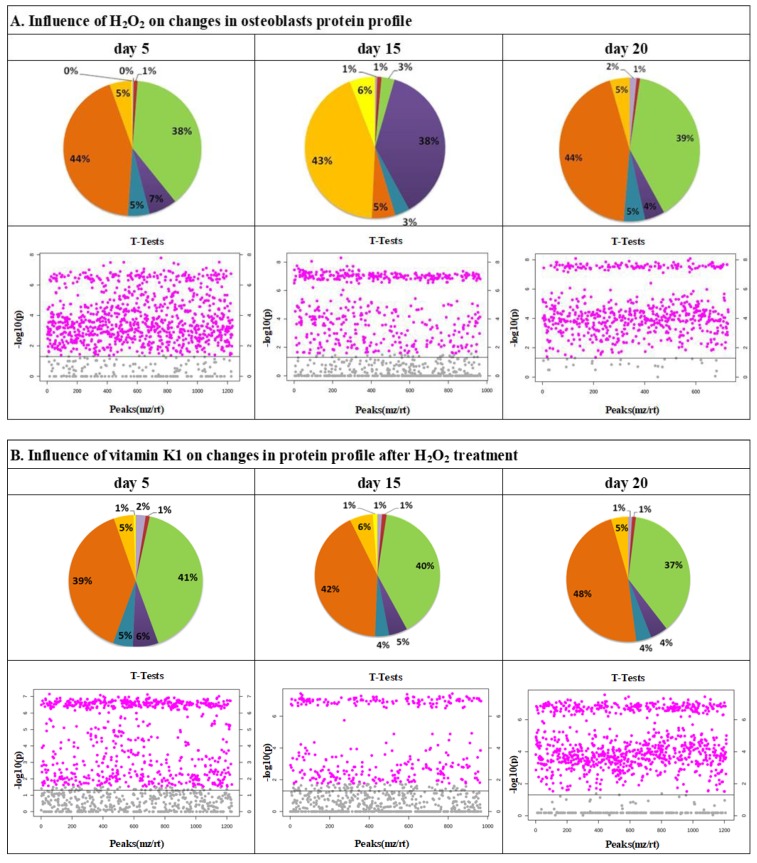

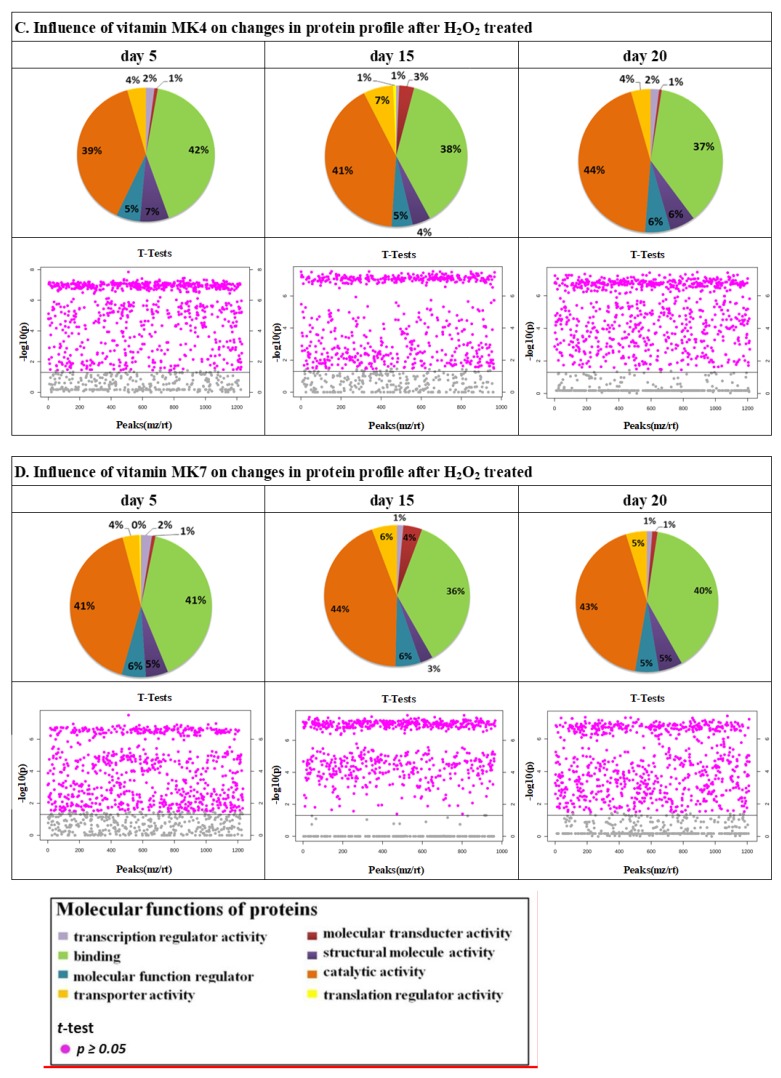
The molecular functions and *t*-test analysis of proteins in osteoblasts treated with H_2_O_2_ (200 μM for 24 h) (**A**) and K1 (**B**), MK4 (**C**), or MK7 (**D**) (10 µM for 5, 15, and 20 days). *p*-values less than 0.05 were considered statistically significant. *p*-values presented on figure are included in Table S4.

Protein levels in H_2_O_2_-treated osteoblasts supplemented with vitamins K1, MK4, and MK7 were compared to H_2_O_2_-treated osteoblasts. The differentially expressed proteins were grouped according to their function, and are presented in Figure 1. In osteoblasts treated with vitamins K1, MK4, and MK7 and H_2_O_2_, the most frequent changes were in the expression of proteins with catalytic activity or protein/DNA binding properties (about 45% and 40% of the total number of altered proteins after vitamin treatment following H_2_O_2_ compared to cells treated with only H_2_O_2_). Significant changes were also detected for proteins with transcription/translation regulator activity (2–6%), regulators of molecular functions (5–6%), signal transducers (1–4%), transporters (4–6%), and structural molecules (3–5%; Figure 1).

In addition, unambiguous statistical analysis of these single proteins showed that vitamins K had the most substantial effect on the expression of proteins with transcription regulator (Table 1) and transporter activity (Table 2). We identified 23 proteins with significantly different expression in osteoblasts treated with vitamin K1 (15, 7, and 11 proteins were significantly different after 5, 15, and 20 days, respectively). We also identified 26 proteins with significantly altered expression in osteoblasts treated with vitamin MK4 (15, 11, and 10 proteins with significantly different expression after 5, 15, and 20 days, respectively). Finally, we detected 21 proteins with expression changes following vitamin MK7 treatment (6, 10, and 10 significantly different proteins after 5, 15, and 20 days, respectively).

In the case of vitamins K1 and MK4, almost all of the changed proteins were downregulated after five days in culture, while in the following days, more and more upregulated proteins were identified. Only vitamin MK7 induced the strong downregulation of protein expression after 15 days. These findings were compared with protein changes induced by H_2_O_2_ alone, where following five days, 11 proteins were upregulated, and only 1 downregulated, while in the next days, more proteins became downregulated at the expense of upregulated proteins (Table 1 and Table 2).

## 3. Discussion

One of the main factors that disrupts osteoblast metabolism is oxidative stress. Oxidative stress is characterized by overproduction of ROS, such as superoxide and hydrogen peroxide, which can cause severe damage to cellular macromolecules such as DNA, proteins, and lipids. Oxidative stress induces a broad spectrum of metabolic responses, including proliferation, growth arrest, differentiation, cell aging, and cell death. These metabolic responses are initiated by activating numerous signaling pathways, such as PI-3K, NF-κB, PLC-c1, p53, HSF, and many MAPKs, in including signal-regulated extracellular kinases (ERK), N-terminal kinase c-Jun (JNK), and p38 [10,25].

The metabolic response that arises from the stimulation or inactivation of particular signaling pathways depends on the type of cells and the severity and duration of stress [26]. It has been demonstrated that redox imbalance is associated with bone remodeling, which enables continuous bone regeneration through the coordinated actions of bone cells such as osteoclasts, osteoblasts, and osteocytes [3]. However, persistent oxidative stress may lead to the development of bone diseases, among which osteoporosis is the most serious [27]. Therefore, it is also believed that oxidative stress is involved in the pathogenesis of bone loss. ROS induce apoptosis of osteoblasts and osteocytes, promoting osteoclastogenesis and inhibiting mineralization and osteogenesis. Increased osteocyte apoptosis also correlates with oxidative stress, shifting bone metabolic processes towards osteoclastogenesis, leading to increased bone remodeling and bone loss.

Additionally, the disturbances in osteoblasts metabolism induced by ROS, as well as other harmful factors can lead to the development of serious diseases, which is also associated with modifications in the bone cell proteome. In vitro studies show that osteoblasts isolated from patients with various bone disease exhibit modified gene expression [28]. Proteomic experiment on rats indicates that osteoporosis development is associated with changes in protein levels, including thioredoxin peroxidase 1, myosin light polypeptide 2, and ubiquitin-conjugating enzyme E2 [29]. Moreover, it has been found in in vivo analysis, that in the case of human bone cancer metastases the cells proteome is more heterogeneous than in the case of normal tissue. The increased levels of proteins involved in cell-cycle progression, DNA damage response, RNA processing, and fatty acid β-oxidation has been observed. Only proteins related to cell adhesion has reduced expression what additionally favors further metastases [30]. The changes in bone cells proteome might be also visible in the serum of patients, what has been demonstrated for multiple myeloma patients, whose changes in enzymes and extracellular matrix glycoproteins level in blood has been correlated with progress of bone disease [31].

According to the dangers of the changes in bone cells proteomic profile, compounds (especially natural compounds) that can protect osteoblasts, including osteoblast metabolic proteins, against the harmful effects, including oxidative stress are currently sought after [32,33,34]. Vitamin K has been shown to significantly reduce oxidative stress in human osteoblasts through reducing ROS production, as well as reducing the level and activity of nonenzymatic and enzymatic antioxidants [35]. However, the effect of vitamin K on the structure and function of osteoblast proteins subjected to oxidative stress during the osteoblast proliferation and differentiation has yet to be investigated. The proteomic data reported here shows, for the first time, that in osteoblasts, the proteins that are protected by vitamins K1, MK4, and MK7 against oxidative stress are mainly proteins with transcription and transport functions. This particularly highlights their role in cell function, communication, and reactions to oxidative stress.

Osteoblasts, that are responsible for bone formation, differentiate from mesenchymal cells. Their differentiation runs in the following three phases: proliferation, extracellular matrix synthesis (maturation), and bone extracellular matrix mineralization [36,37]. Cells transformations between these phases are precisely coordinated due to the activity of numerous transcription regulators that are expressed selectively and at high levels in osteoblasts [38]. Transcription factors such as Runx2, osterix, and β-catenin are essential for osteoblast differentiation. Runx2 directs mesenchymal cells to an osteoblastic lineage and leads them to differentiation to preosteoblasts. Then, in cooperation with osterix and β-catenin directs them to immature osteoblasts. If these transcription factors are not inhibited at this stage osteoblasts cannot change into mature cells [39]. For further osteoblasts differentiation is also needed other transcription factors, including Msx1, Msx2, Dlx5, Dlx6, Twist, AP1(Fos/Jun), Knox-20, Sp3, or ATF4 [39,40]. However, disturbances of transcription regulators/factors in osteoblasts are strictly associated with bone formation abnormalities and can be closely linked to diseases development [41].

One of the proteins with transcription regulator activity that expression has been significantly changed in this study is Src substrate cortactin (Q14247). The results obtained here show that vitamins MK4 and MK7 cause an increase in the level of Src substrate cortactin in osteoblasts treated with H_2_O_2_ during the proliferation period (day 5 of the experiment), indicating an increase in cell motility [42], characteristic for the proliferation process of osteoblasts. In the following days of the experiment, no statistically significant changes in the concentration of this protein were observed, likely related to the fact that osteoblast motility decreases when cells begin the process of differentiation [43]. The role of Src substrate cortactin in the proliferation process is cortactin phosphorylation. Phosphorylated cortactin can then interact with actin, leading to the remodeling of the actin skeleton. This remodeling involves the loosening of intercellular junctions and cell dispersal [44], which may promote bone reconstruction and regeneration.

Bone mass and structure depend primarily on normal cell proliferation, differentiation, and apoptosis [45]. Osteoblasts are responsible for osteogenesis and mineralization both in the case of bone formation and its growing but also in its damage reparation. In both cases, factors responsible for osteoblasts proliferation, providing a sufficient amount of cells and differentiation, ensuring the functionality of mature cells, are equally important as pro- and antiapoptotic molecules. Antiapoptotic factors do not allow cell death during their differentiation, what significantly increases bone formation [46], while proapoptotic molecules, by eliminating damaged cells, prevent carcinogenesis and indirectly provide the building material for building a new bone [47]. On the other hand, harmful factors (including electrophilic molecules) as well as anti-inflammatory drugs have been shown as a molecules that in low concentration stimulate proliferation and differentiation of osteoblasts, however, in high concentration not only inhibits cells proliferation and differentiation but also causes apoptosis [48,49].

Mentioned processes of osteoblasts proliferation, differentiation, and apoptosis are within the cell regulated by many signaling pathways, including cell division cycle and apoptosis regulator 1 (CARP-1 and Q8N163) [50,51]. This protein regulates cell growth and differentiation in the parathyroid hormone pathway (PTH/PTHR1) [52] and in the Wnt-dependent canonical signaling pathway, including β-catenin [53]. These pathways are responsible for bone mass increase and osteoblast apoptosis [54,55]. Under physiological conditions, during the process of proliferation, cells should maintain a high level of CARP-1 protein, which decreases only during osteoblast differentiation [48]. The observed decrease in the expression of this protein, increased as a result of oxidative stress caused by hydrogen peroxide, caused by vitamins K may indicate the participation of these vitamins in cell cycle regulation and CARP-1 mediated apoptosis. These results are supported by the studies of cancer cells (hepatocellular carcinoma cells) in which vitamins K also significantly reduced CARP-1 expression, thereby eliminating modified and dangerous cells [56].

One of the main group of proteins significantly changed by vitamins K are transporters. The role of these molecules has been widely described in the literature with a particular focus on participation in the regulation of bone remodeling [57]. The activity of transmembrane transporters provides the delivery of molecules necessary for cells development, including sodium-dependent vitamin C transporter [58], as well as ensures bone cells proper communication with the organism, as in the case of norepinephrine or taurine transporter [59,60]. Additionally, it has been found that transporters located in osteoblasts membranes can receive nerve-derived signals, including adrenergic, glutaminergic, serotoninergic, dopaminergic, and sensory nature [57]. The proper activity of these transporters plays a direct role in bone homeostasis also by inhibiting osteoclastogenesis. It has been shown, among others, that the activity of the glutamate transporter is necessary especially in mature osteoclasts, where inhibition of the glutamate transporter increased the number of bone resorptive pits and significantly reduced the number of osteoclasts [61].

We also observed that osteoblasts on day 5 of hydrogen peroxide exposure displayed an increase in the level of proteins with transporter activity, including importin-7 and -9 (O95373, Q96P70, Q14974.). Treatment with vitamins K1 and MK4 significantly prevented these modifications. The primary role of importin is intracellular transport, and reduced levels of importin-9 are conducive to inhibiting osteogenesis [62], as this protein is involved in osteoblast differentiation by translocation of actin to the nucleus, thereby limiting actin polymerization [63]. The presence of actin in the nucleus is associated with the overexpression of osteogenic proteins such as osterix and osteocalcin in the Runx2-dependent pathway, which causes intensive osteoblastogenesis in vitro and local bone formation in vivo [62]. However, the observed reduction of these proteins level by vitamin K in H_2_O_2_-treated osteoblasts may indicate that—by counteracting the harmful effects of oxidative stress—group K vitamins may partially block signals that stimulate cells to remodel and repair bone.

In another example, vitamins K1 and MK4 strongly inhibited the H_2_O_2_-induced increase of vacuolar protein sorting 35 (VPS35 and Q96QK1). VSP35 is a protein present in both osteoblasts and osteoclasts [64], and as a part of retromer, is involved in the transfer of signaling molecules PTH1R, Wntless, and RANK from endosomes to the Golgi apparatus, where it initiates bone remodeling [65]. In addition, VPS35 is thought to be essential for bone formation because mice lacking VSP35 exhibit lower bone mass [65]. Therefore, blocking the hydrogen peroxide-induced increase in VSP35 expression through treatment with vitamins K1 and MK4 prevents overproduction of bone mass crucial for bone tissue homeostasis.

Osteoblast apoptosis plays a key role in skeletal development and bone maintenance. As evidence of this, increased osteoblast apoptosis is observed in the pathogenesis of many bone diseases, contributing to bone loss [66]. To maintain cell density, osteoblasts are sensitive to continuous signals from an external beneficial microenvironment. However, when these signals are reduced as a result of injury or illness, cells lacking stimulation will undergo apoptosis. One of the regulators of apoptosis in osteoblasts is the proapoptotic Bax protein, which belongs to the Bcl-2 family [67]. This protein is found in the cytosol, and after induction of apoptotic signaling, it moves towards and binds to the mitochondrial membrane. The proapoptotic mechanism of action of Bax is based on the interaction with the anion channel, dependent on the mitochondrial voltage canal of VDAC, and causes its opening. Additionally, Bax may form oligomeric pores in the cell membrane, causing the release of cytochrome c and other proapoptotic factors from mitochondria, resulting in the activation of caspases. Bax activation is stimulated by various factors, including hydrogen peroxide [68]. However, the results of this study show that all tested forms of vitamin K (K1, MK4, and MK7) are effective in protecting osteoblasts against the toxic and proapoptotic effects of H_2_O_2_ by reducing Bax levels as previously found in Western blot analysis [69].

An important aspect of bone formation and mineralization is the proper transport of ions across the cell membrane, especially those necessary for the mineralization process, such as calcium and phosphate ions. Oxidative stress has been shown to reduce the activity of ion channels [70]. However, the results described here demonstrate a protective effect of vitamin MK7 on H_2_O_2_-treated osteoblasts at a late stage of differentiation since, on the 20th day of treatment, a significant increase in calcium transporting ATPase expression was observed (PMCA and P20020). The osteoblast cell membrane contains two main proteins responsible for calcium transport: the Na^+^/Ca2^+^ exchanger (NCX; located along the surface of the osteoblast near the osteoid), and PMCA (located on the opposite side of the cell from the osteoblast). Both channels are involved in the regulation of calcium levels in osteoblasts and are activated during differentiation [71]. The NCX protein is associated with bone mineralization. In contrast, PMCA is responsible for calcium homeostasis regulated by signaling pathways, that is, it is responsible for calcium transport from the cytoplasm to the extracellular space, and thus positively regulates bone mineralization by absorption of calcium from the digestive system [71,72]. In this aspect, stimulation of PMCA expression by vitamin MK7 promotes normal bone mineralization even under conditions of oxidative stress.

## 4. Materials and Methods

### 4.1. Cell Culture and Treatment

All experiments were carried out using a normal human fetal osteoblast cell line (hFOB 1.19) obtained from American Type Culture Collection (ATCC, Manassas, VA, USA). The cells were cultured in growth medium (DMEM/F12) supplemented with G418 (0.3 mg∙mL^−1^), penicillin (100 U∙mL^−1^), streptomycin (100 μg∙mL^−1^), and FBS (10%), and incubated at 34 °C in 5% CO_2_. The process of osteoblast differentiation was induced by increasing the temperature to 38 °C and changing the growth medium to a mineralization medium, which was prepared by supplementing the growth medium with ascorbic acid (0.05 mg·mL^−1^), sodium β-glycerophosphate (10 mM), and dexamethasone (100 nM) [73]. Under these conditions, the cells were cultured for 5, 15, and 20 days. The incubation time was chosen according to various osteoblast differentiation phases: proliferation, extracellular matrix synthesis (maturation), and bone extracellular matrix mineralization, which are characterized by different ALP activity [36,37].

To study the effects of vitamin K1, MK4, and MK7 on the differentiation of hFOB 1.19 osteoblasts under oxidative conditions, the cells were treated with H_2_O_2_ as described by Arai et al. (2007) [11] with minor modifications. Osteoblasts were seeded at a high-density to ensure close cell–cell contact within the 90 mm plate. After 24 h, 11 days, and 16 days, the cells were treated with H_2_O_2_ (200 μM) and cultured for the next 24 h. The osteoblasts were then washed with fresh medium to remove H_2_O_2_, and vitamin K1, MK4, and MK7 were added to the cells at a concentration of 10 µM. After 5, 15, and 20 days, osteoblasts were collected for analysis.

### 4.2. SDS-PAGE and In-Gel Digestion

All cells were collected after treatment with H_2_O_2_, vitamins K1, MK4, and MK7, and lysed in Tris-HCl buffer (50 mM, pH 7.5) containing SDS (0.1%), Triton X-100 (1%) and a protease inhibitor cocktail. Protein concentrations were determined by the Bradford method, and the volume of the sample containing 25 μg of protein was mixed 1:1 with Laemmle buffer containing 2-mercaptoethanol (5%), and boiled for 5 min (min). The proteins were then separated on 10% Tris-Glycine SDS-PAGE gels. Following electrophoresis, the gels were fixed in methanol: acetic acid: water (4:1:5) for 1 h and stained overnight with Coomassie Brilliant Blue R-250. All lanes were excised out of the gel. Then, proteins were reduced and alkylated with DTT (10 mM) and IAA (50 mM), respectively. Next, proteins were in-gel digested overnight with sequencing grade trypsin (Promega, Madison, WI, USA). The peptide mixture was extracted from the gel fractions and dried [74].

### 4.3. Liquid Chromatography-Mass Spectrometry (LC-MS)/MS Analysis

The dried peptide fraction was dissolved in 50 µL of acetonitrile (5%) with formic acid (0.1%), and 5 µL of the sample was loaded onto a PepMap RSLC capillary analytical C18 column (150 mm × 75 mm, 2 μm particle size; Dionex, LC Packings, Idstein, Germany) at a flow rate of 0.3 µL/min using an Ultimate 3000 (Dionex, Idstein, Germany). To analyze the samples, a gradient elution process was performed, starting at 3 min, and ramped to 60% Buffer B (90% acetonitrile + 0.03% FA) over 60 min [75]. The eluted peptides were analyzed using a Q Exactive HF mass spectrometer with a nanoelectrospray ionization source (ESI; Thermo Fisher Scientific, Bremen, Germany). The data was acquired with the Xcalibur software (Thermo Fisher Scientific, Bremen, Germany).

### 4.4. Protein Identification and Label-Free Quantification

The processing of the raw data generated from the LC-MS/MS analysis was performed using Proteome Discoverer 2.0 (Thermo Fisher Scientific, Bremen, Germany) and Sequest HT (SEQUEST HT algorithm, license Thermo Scientific, registered trademark University of Washington, Seattle, WA, USA). The following search parameters were used to identify proteins: peptide mass tolerance—10 ppm; MS/MS mass tolerance—0.02 Da; mass precision—2 ppm; missed cleavages—up to 2; minimal peptide length—6 amino acids; and minimal number of identified unique peptides for single protein—2 peptides. Input data were searched against the UniProtKB-SwissProt database (taxonomy: *Homo sapiens*, release 2018-04). Label-free quantification was based on the peak area for the individual peptides and proteins. Protein grouping was performed according to molecular function using the Gene Ontology database available in the free available Panther Classification System (http://pantherdb.org).

### 4.5. Statistical Analysis

The analysis of each sample was performed in three independent experiments. Results from individual protein label-free quantification were log-transformed and analyzed using standard statistical analysis methods, including the one-way Student’s *t*-test for multiple comparisons, with MetaboAnalyst 4.0 software (https://www.metaboanalyst.ca). *p*-values less than 0.05 were considered statistically significant.

## 5. Conclusions

The results obtained in this study demonstrated that vitamins K1, MK4, and MK7 could prevent H_2_O_2_-induced changes in the proteome profile of osteoblasts at different stages of proliferation and differentiation. This effect was primarily mediated by altering the expression of transcription regulators and transporters. Therefore, vitamin Ks could effectively regulate bone formation, remodeling, and mineralization.

## Figures and Tables

**Table 1:** The list of proteins with transporter activity that expression was significant changed in osteoblasts treated with H_2_O_2_ (200 μM for 24 h) and K1, MK4, or MK7 (10 µM for 5, 15, and 20 days). Arrows indicate the direction of statistically significant changes for individual proteins (↑—upregulation, ↓—downregulation). Mean value of three independent experiments with standard deviation (SD) for each protein are presented in Tables S1–S3.

ID	Protein Name	H_2_O_2_ vs. Control			H_2_O_2_ + K1 vs. H_2_O_2_			H_2_O_2_ + MK4 vs. H_2_O_2_			H_2_O_2_ + MK7 vs. H_2_O_2_		
		Days											
		5	15	20	5	15	20	5	15	20	5	15	20
P05023	Na/K-transporting ATPase subunit α1				↓			↓					
P38606	v-type proton ATPase catalytic subunit A		↓	↓	↓	↑	↑	↓	↑				
O14983	Sarcoplasmic/endoplasmic reticulum calcium ATPase 1		↑			↑			↑			↓	
P20020	Plasma membrane Ca-transporting ATPase			↓									↑
O95373	Importin-7	↑			↓			↓					
Q96P70	Importin-9	↑			↓			↓					
Q14974	Importin subunit beta-1	↑			↓								
P55060	Exportin-2	↑			↓		↑	↓		↓			
Q96QK1	Vacuolar protein sorting-associated protein35	↑	↑		↓	↓		↓	↓			↓	
Q15758	Neutral amino acid transporter B(0)		↑	↓	↓	↑	↑	↓	↓	↓		↓	
Q8WVM8	Sec1 family domain-containing protein 1	↑	↓	↓	↓	↑	↑		↑			↓	↑
O60518	Ran-binding protein 6	↑			↓		↑	↓		↓			↓
Q00325	Phosphate carrier protein, mitochondrial					↑	↓			↑			↑
Q00765	Receptor expression-enhancing protein 5			↓			↓			↓			↓
Q02978	Mitochondrial 2-oxoglutarate/malate carrier protein			↓			↓	↓	↑	↑		↑	↑
Q07812	Bax			↑			↓			↓			↓
P60468	Protein transport protein Sec61 subunit beta	↑						↓	↑				
O75746	Ca-binding mitochondrial carrier protein Aralar1		↑						↑			↓	
Q8N4V1	Membrane magnesium transporter 1								↑				
Q99523	Sortilin		↑									↓	↑

**Table 2:** The list of proteins with transcription regulator activity that expression was significant changed in osteoblasts treated with H_2_O_2_ (200 μM for 24 h) and K1, MK4, or MK7 (10 µM for 5, 15, and 20 days). Arrows indicate the direction of statistically significant changes for individual proteins (↑—upregulation, ↓—downregulation). Mean value of three independent experiments with standard deviation (SD) for each protein are presented in Tables S1–S3.

ID	Protein Name	H_2_O_2_ vs. Control			H_2_O_2_ + K1 vs. H_2_O_2_			H_2_O_2_ + MK4 vs. H_2_O_2_			H_2_O_2_ + MK7 vs. H_2_O_2_		
		Days											
		5	15	20	5	15	20	5	15	20	5	15	20
O75475	PC4 and SFRS1-interacting protein	↑			↓					↑	↓		
Q14247	Src substrate cortactin	↑			↓			↑			↑		
Q8N163	Cell cycle and apoptosis regulator protein 1	↑			↓			↓			↓		
B2RPK0	Putative high mobility group protein B1				↓			↓			↓		
P13489	Ribonuclease inhibitor	↓			↑			↑			↑		
E9PA3	Nascent polypeptide-associated complex					↓		↑			↑	↓	
Q13263	Transcription intermediary factor 1-β		↑				↑		↓	↓		↓	↑
Q07666	KH domain-containing, RNA-binding, signal transduction-associated protein 1						↓			↓			↓

## Supplementary Materials

The following are available online, Table S1: The level of proteins with transporter or transcription regulator activity that expression was significant changed in osteoblasts treated with H2O2 (200μM for 24h) and vitamins K1, MK4, or MK7 (10 µM for 5 days), Table S2: The level of proteins with transporter or transcription regulator activity that expression was significant changed in osteoblasts treated with H2O2 (200μM for 24h) and vitamins K1, MK4, or MK7 (10 µM for 15 days), Table S3: The level of proteins with transporter or transcription regulator activity that expression was significant changed in osteoblasts treated with H2O2 (200μM for 24h) and vitamins K1, MK4, or MK7 (10 µM for 20 days), Table S4: The p values of proteins expression obtained basing on label-free quantification in osteoblasts treated with H2O2 (200μM for 24h) and vitamins K1, MK4, or MK7 (10 µM for 5, 15, 20 days).
